# A Comparison of the Antibacterial Efficacy of Carbohydrate Lipid-like (Thio)Ether, Sulfone, and Ester Derivatives against *Paenibacillus larvae*

**DOI:** 10.3390/molecules28062516

**Published:** 2023-03-09

**Authors:** Veronika Šamšulová, Mária Šedivá, Juraj Kóňa, Jaroslav Klaudiny, Monika Poláková

**Affiliations:** 1 Department of Organic Chemistry, Faculty of Science, Palacký University, 17. Listopadu 12, 779 00 Olomouc, Czech Republic; 2 Center for Glycomics, Institute of Chemistry, Slovak Academy of Sciences, Dúbravská Cesta 9, 84538 Bratislava, Slovakia; 3 Medical Vision, Civic Research Association, Záhradnícka 4837/55, 82108 Bratislava, Slovakia

**Keywords:** carbohydrate esters, carbohydrate (thio)ethers, fatty acid, lauric acid, monolaurin, 10-HDA, royal jelly, antibacterial activity, American foulbrood

## Abstract

*Paenibacillus larvae* is the causative agent of American foulbrood (AFB), the most serious bacterial disease affecting developing honeybee larvae and pupas. In this study, a library of 24 (thio)glycosides, glycosyl sulfones, 6-*O*-esters, and ethers derived from d-mannose, d-glucose, and d-galactose having C10 or C12 alkyl chain were evaluated for their antibacterial efficacy against two *P. larvae* strains. The efficacy of the tested compounds determined as minimal inhibitory concentrations (MICs) varied greatly. Generally, dodecyl derivatives were found to be more potent than their decylated analogs. Thioglycosides were more efficient than glycosides and sulfones. The activity of the 6-*O*-ether derivatives was higher than that of their ester counterparts. Seven derivatives with dodecyl chain linked (thio)glycosidically or etherically at C-6 showed high efficacy against both *P. larvae* strains (MICs ranged from 12.5 μM to 50 μM). Their efficacies were similar or much higher than those of selected reference compounds known to be active against *P. larvae*—lauric acid, monolaurin, and honeybee larval food components, 10-hydroxy-2-decenoic acid, and sebacic acid (MICs ranged from 25 μM to 6400 μM). The high efficacies of these seven derivatives suggest that they could increase the anti-*P. larvae* activity of larval food and improve the resistance of larvae to AFB disease through their application to honeybee colonies.

## 1. Introduction

American foulbrood (AFB) is initiated through the infection of young honeybee larvae with food contaminated by spores of the Gram-positive bacterium *Paenibacillus larvae* [1,2,3]. The pathological development of larval infections is associated with spore germination followed by the massive multiplication of the vegetative cells of the bacteria in the larval midgut. During this process, various substances are produced by pathogen cells, some of which help the cells penetrate through the intestinal epithelium into the larva hemocoel [4,5]. Here, the cells further multiply, leading to the death of the larva (or pupa), its decomposition, and the formation of billions of new spores. The spores are transmitted by worker bees into the food of other larvae, resulting in more and more of the latter becoming sick and dying, which finally causes the collapse of a diseased colony [6]. AFB disease is highly contagious and it spreads among colonies in several ways [7,8,9]. Its outbreaks are quite frequent in honeybee populations, causing large annual financial losses for beekeepers worldwide as well as for farmers dependent on crop pollination.

Honeybee colonies differ in resistance to AFB [3,6]. This resistance is associated with the immunity of individual larvae [10,11,12] and the social immunity mediated by many worker bees [13,14,15]. One of the immune social mechanisms is probably associated with the antibacterial properties of larval food [royal (RJ), worker (WJ), and drone (DJ) jelly] [16,17]. The food is produced by genetically heterogeneous populations of nurse bees in honeybee colonies and varies in constitutive contents of various antibacterial substances. These include proteinaceous compounds [18,19,20] and different derivatives of C8–C12 fatty acids (hydroxy and dicarboxylic) [21,22,23] many of which exhibit anti-*P. larvae* activity. These are peptide defensin1 [24,25], protein apalbumin2a [26], major RJ fatty acid 10-hydroxy-2-decenoic (10-HDA) [16], and abundant RJ acids sebacic [27] and 2-decene-1,10-dioic acids [28].

Beekeepers use several strategies to control AFB. These include various preventive precautions and some treatments [3,29,30] including the application of antibiotics to colonies [31,32]. Due to the appearance of resistant strains [33,34] and the possible contamination of honeybee products with them [35,36,37], their use was banned in many countries including EU member states. Instead, radical methods involving the burning of diseased colonies and contaminated hive materials are applied in these countries [6,38]. Other approaches for controlling AFB are therefore being investigated. These are based on the application of anti-*P. larvae* active substances including natural ones such as essential oils, plant extracts, propolis (its components), fatty acids, and probiotic bacteria [38,39,40,41,42], synthetic compounds such as indol analogs [43], and bacteriophages [44].

Fatty acids and some of their derivatives represent a large group of antibacterial substances. It has been reported that different fatty acids such as saturated C10–C14, monounsaturated C14, C16, C18, and some polyunsaturated C18–C22 fatty acids exhibit anti-*P. larvae* activity. The most active among them are lauric, myristoleic, palmitoleic [45], undecanoic, and homo-γ-linolenic acids [46]. Among the fatty acid derivatives, monolaurin, a glycerol monoester of lauric acid, was found to be effective against *P. larvae* [47]. Furthermore, it has also been shown that carbohydrate fatty acid esters are antibacterially active but mainly against Gram-positive bacteria [48,49,50]. The ester bond in these derivatives can, however, be cleaved by esterases in cells or guts leading to fatty acid and inactive sugar [51]. To avoid such hydrolysis, the ester linkage can be replaced by a (thio)ether bond which is resistant to esterases [52]. The activity of various ether derivatives has been found to be dependent on the alkyl chain length and the stereochemistry of the carbohydrate unit. The most efficient derivatives were those bearing decyl and dodecyl alkyl chains [52,53,54]. To the best of our knowledge, the inhibition effects of carbohydrate (thio)ether and ester derivatives against *P. larvae* have not been explored yet and this inspired us to perform the investigation presented here.

In this work, various lipid-like carbohydrate derivatives were studied for their antibacterial effects against two *P. larvae* strains possessing the ERIC I and ERIC II genotypes (ERIC- Enterobacterial Repeating Intergenic Consensus sequences). Only these two genotypes occur in field isolates of *P. larvae.* AFB disease caused by strains of the distinct genotypes exerts different time progression in infected larvae and in whole colonies, which is manifested by partially different symptoms in colonies [6,55]. The tested derivatives included (1) decyl and dodecyl (thio)glycosides, and glycosyl sulfones derived from d-mannose, d-glucose, and some from d-galactose; (2) glycosylated fatty acids (10-HDA, capric, and lauric acids) with d-mannose and d-glucose; and (3) 6-*O*-ethers and esters (with C12 alkyl chain) of methyl α-d-glycosides from d-mannose and d-glucose. Their antibacterial efficacies against *P. larvae* were determined and compared to reference compounds including fatty acids (lauric, sebacic, and 10-HDA) and their derivatives (monolaurin and monomethyl sebacate). The relations among chemical structures of the compounds and their anti-*P. larvae* efficacies were evaluated. Several new efficient compounds against the pathogen were identified. The potential of the most efficient compounds to increase the anti-*P. larvae* activity of larval food and improve the protection of larvae against AFB through their application (as individual substances) to honeybee colonies is discussed.

## 2. Results and Discussion

### 2.1. Synthesis

**Figure 1:**
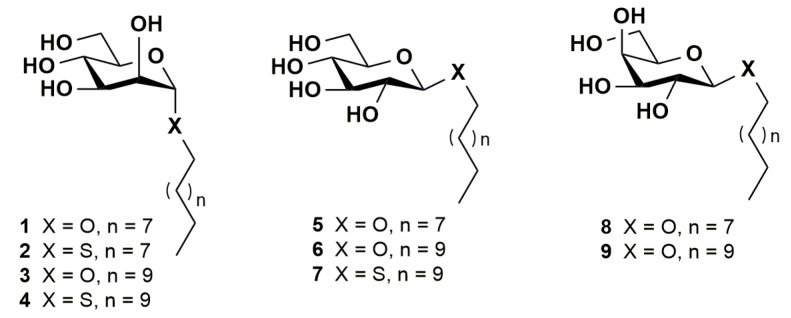
The previously synthesized nonionic glycolipids.

**Scheme 1:**
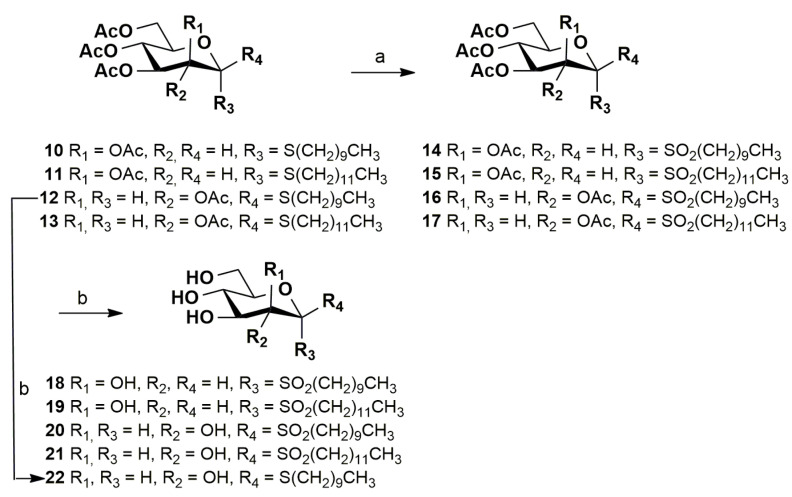
Synthesis of the sulfones **18**–**21** and thioglucoside **22**. *Reagents and conditions*: (**a**) *m*CPBA, CH_2_Cl_2_, 2 h, rt, 85% for **14**, 83% for **15**, 75% for **16**, 80% for **17**; (**b**) MeONa, MeOH, 16 h, rt, 82% for **18**, 80% for **19**, 81% for **20**, 83% for **21,** 75% for **22**.

Glycolipid mimetics having C10 and C12 alkyl chains attached to d-mannose (**1**–**4**), d-glucose (**5**–**7, 22**), and d-galactose (**8**, **9**), in the form of *O*- and *S*- glycosides (Figure 1, Scheme 1) and sulfones (Scheme 1) were designed as the first set of amphiphilic structures to be examined. These derivatives are easily available through a short sequence, i.e., through glycosylation of the corresponding (thio) alcohols with per-*O*-acetylated glycosyl donors, followed by saponification of the acetyl protective groups; most had been previously prepared [52,53,56].

In the synthesis of compounds **18**–**21,** the highly efficient oxidation of glycosidic sulfur with *m*CPBA yielding the corresponding protected sulfones preceded a saponification step. Decyl thioglucoside **22** was prepared by deacetylation of the compound **12** (Scheme 1).

**Scheme 2:**
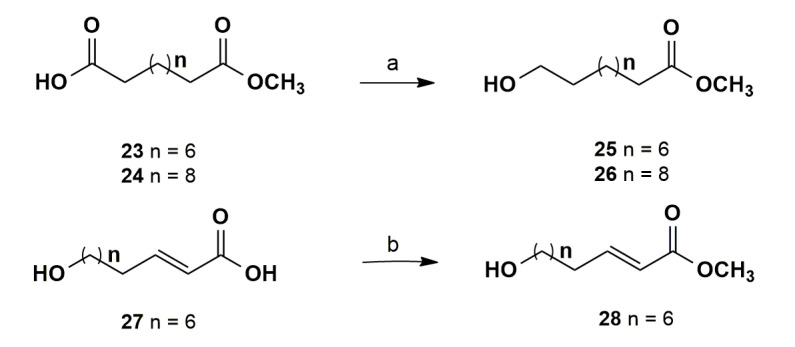
Synthesis of the glycosyl acceptors **25**, **26,** and **28.** *Reagents and conditions*: (**a**) 1M BH_3_.THF, THF, 16 h, rt, 78% for 25, 80% for 26; (**b**) MeOH, Amberlite IR 120, H^+^ form, 96 h, rt, 73%.

Glycosyl derivatives of fatty acids (capric, lauric, and 10-HDA) linked via their ω-hydroxyl group to d-mannose and d-glucose were synthesized as follows. First, the glycosyl acceptors **25**, **26**, and **28** (ω-hydroxylated fatty acid methyl esters) were prepared in one step (Scheme 2). The **25** and **26** were obtained through a reduction of the free carboxylic function of the corresponding dicarboxylic acid monomethyl esters (**23** and **24**) with BH_3·_THF [57]. Acceptor **28** was prepared in a moderate yield via the esterification of **27** (10-HDA).

**Scheme 3:**
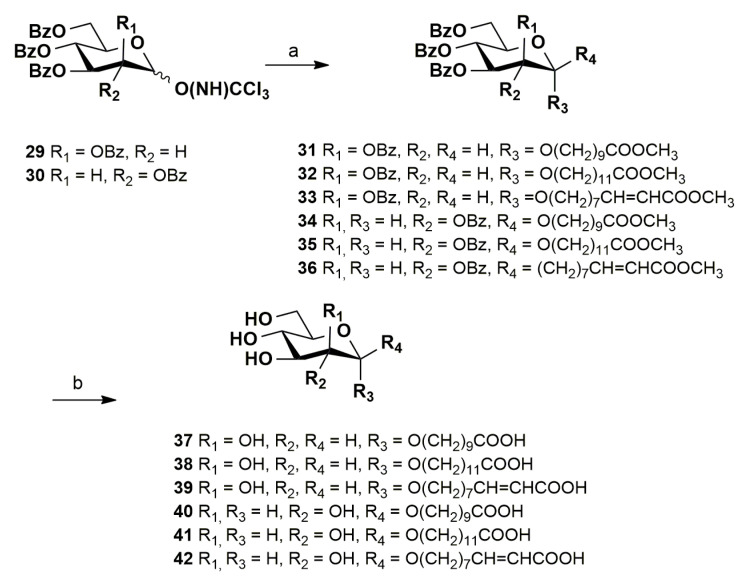
Synthesis of ω-*O*-glycosylated fatty acids **37**–**42**. *Reagents and conditions*: (**a**) **25**, **26**, or **28**, TMSOTf, CH_2_Cl_2_, 20 min, rt, 71% for **31**, 79% for **32**, 85% for **33**, 71% for **34**, 65% for **35**, 71% for **36**; (**b**) MeONa, MeOH, 1 h, rt, then LiOH, 1h, rt, 92% for **37**, 91% for **38**, 81% for **39**, 81% for **40**, 72% for **41**, 75% for **42**.

These acceptors were reacted with per-*O*-benzoylated imidates **29 [58]** and **30 [59]** serving as glycosyl donors. The coupling reaction was promoted by TMSOTf and provided the conjugates **31**–**36** (Scheme 3). Then, all ester protective groups were easily removed following the Zemplen protocol (MeONa, MeOH, debenzoylation) in the first step, and their subsequent treatment with LiOH (fatty acid de-esterification) provided the target derivatives **37**–**42** in satisfactory overall yields.

**Scheme 4:**
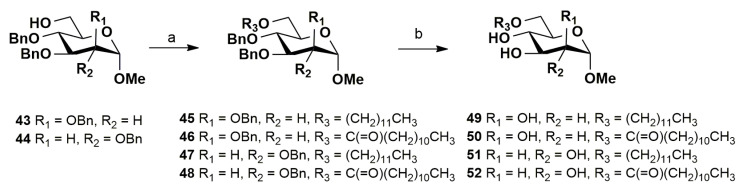
Synthesis of 6-*O*-substituted glycosides **49**–**52**. *Reagents and conditions*: (**a**) C_12_H_25_Br, NaH, DMF, 16 h, rt, 75% for **45**, 94% for **47**; C_11_H_23_COCl, DMAP, pyridine, 24 h, rt, 79% for **46**, 94% for **48**; (**b**) Pd/C, H_2_, MeOH, 4 h, rt, 79% for **49**, 72% for **50**, 82% for **51**, 98% for **52**.

A synthetic sequence leading to the 6-*O*-ether and ester derivatives of methyl α-d-glycosides is shown in Scheme 4. Compounds **43** and **44** were synthesized using a strategy based on a selective deprotection of the least sterically hindered primary 6-OH group of the corresponding glycosides. In the case of mannoside **43 [60]**, the three-step sequence started with per-*O*-benzylation, followed by the selective acidic deprotection of the C-6 position by TFA/AcOH and the saponification of 6-*O*-acetyl derivative. Glucose unit **44 [61,62]** was also obtained over three steps, namely through tritylation followed by benzylation, and finally detritylation.

The direct etherification or esterification of **43** and **44** with the corresponding alkyl bromides or acyl chlorides yielded the 6-*O*-subtituted glycosides **45**, **46 [49]**, **47**, and **48 [49]**. In the last step, the removal of benzyl groups by catalytic hydrogenation provided the desired 6-*O*-ethers **49** and **51 [49]** and 6-*O*-esters **50 [49]** and **52 [49]**.

### 2.2. Efficacy of Derivatives against P. larvae

**Figure 2:**
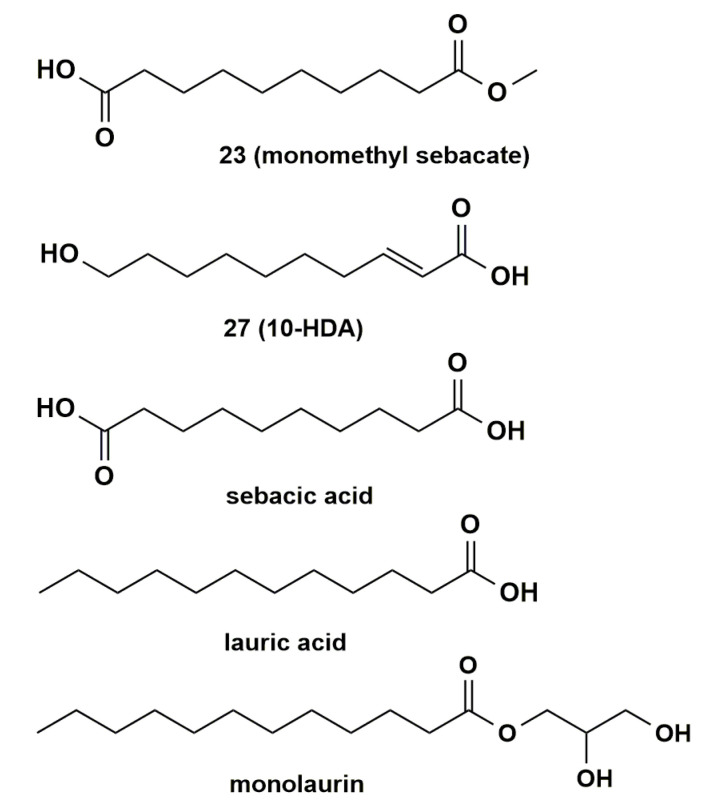
The reference compounds.

The antibacterial efficacies of 24 glycolipid mimetics derived from d-mannose, d-glucose, and d-galactose, five reference compounds (Figure 2), and two antibiotics (ciprofloxacin and tylosin tartrate) were evaluated against two *P. larvae* strains CCM 4483 (ERIC I genotype) and CCM 4486 (ERIC II genotype). The results are summarised in Table 1.

The results showed that the derivatives exhibited a similar antibacterial activity against the *P. larvae* strains of ERIC I and ERIC II genotypes. Maximal 2-fold differences in activity were observed for nine out of fourteen decyl and dodecyl (thio)glycosides derivatives (**1**–**9**, **18**–**22**). These were more effective against the *P. larvae* CCM 4486 than the *P. larvae* CCM 4483 strain. Similar compounds having the aglycone alkyl chain capped with the carboxylic group (**37**–**41**) were inactive against both strains in the tested range of activity. The compounds with a dodecyl chain attached to the saccharide C-6 position by either an ether or ester linkage (**49**–**52**) showed the same activity against both strains. The findings suggest that the differences in activity against the strains of different ERIC genotypes occurring at some derivatives were likely caused by a genetic variability of the tested strains and not by the ERIC genotype of *P. larvae*.

Out of 14 alkyl (thio)glycosides and sulfones, the compounds having a dodecyl aglycone were more active than decylated analogs. The effect of the aglycone length was more profound in glucosides and mannosides than in galactosides. Mannosides were generally more potent than glucosides. Among the decyl glycosides, thiomannoside **2** was the most efficient (MICs 100 μM and 50 μM for individual *P. larvae* strains). The most efficient dodecyl derivative was thiomannoside **4** (MICs 25 μM and 12.5 μM), which represented the most potent derivative of all the tested compounds.

The thioglycosides (**2**, **4**, **7**, **22**) were slightly more efficient than their *O*-analogs (**1**, **3**, **5**, **6**). The oxidation of the glycosidic sulfur providing sulfones **18**–**21** led to a decrease in the inhibitory activity of the compounds.

Galactosides **8** and **9** showed a low antibacterial activity, confirming previous observations [52] that galacto-based derivatives inhibited some Gram-positive bacteria significantly weaker than analogous mannosides and glucosides. Therefore, no other galactose derivatives were studied.

Six glycosides **37**–**42** having an alkyl chain capped with an acid (capric, lauric, and 10-HDA) were inactive in the tested MIC range. This suggests that ω-glycosylation of the fatty acids was detrimental to their inhibitory activity against *P. larvae*. From another point of view, the termination of the alkyl aglycone of the *O*-glycosides with a carboxyl group resulted in a loss of inhibitory activity. This confirms that only a derivative with an amphiphilic nature comprising a hydrophilic (carbohydrate) moiety at one end and simultaneously a hydrophobic (aliphatic alkyl chain) moiety at the other end maintains the antibacterial activity.

The derivatives having an alkyl chain attached to the saccharide C-6 position showed high efficiency. However, their potency was slightly affected by a linkage (ether vs. ester) that connects the hydrophobic unit with the saccharide. Methyl 6-*O*-dodecyl α-d-glycosides **49** and **51** were slightly more potent against both *P. larvae* strains than the 6-*O*-acylated analogs **50** and **52**.

In summary, an evaluation of the library of synthetic glycolipid mimetics revealed that the derivatives having alkyl units (thio)etherically linked either at the saccharide C-1 or the C-6 position exhibited a higher antibacterial effect than the corresponding C-6 esters or the C-1 ethers capped with a carboxylic group.

The antibacterial activity of five reference compounds was examined in this study. Two of them, lauric acid and monolaurin, are known to be active against *P. larvae*. An agar diffusion method previously showed that lauric and myristoleic acids were the most active fatty acids against *P. larvae* among 38 different saturated and unsaturated fatty acids [45]. Lauric acid was found to be the second most efficient compound among 13 different natural compounds in tests with 10 *P. larvae* strains (MICs at individual strains were 25 µg or 50 µg/mL) [63]. The high efficacy of monolaurin (MIC 62.2 µg/mL) against four *P. larvae* strains has also been demonstrated in a previous study [47]. The MICs of lauric acid and monolaurin determined in this work were 2.5–5 and 4.5–9 times lower, respectively than those mentioned above. This could be explained by the use of a cultivation medium with a lower pH in our microdilution tests. An increase in antibacterial efficacy by lowering the pH has already been observed for some medium-chain fatty acids including the 10-HDA [16,64,65]. The lauric acid and monolaurin inhibited *P. larvae* with the same efficiency as derivatives **7**, **50**, and **52**, but twice less than derivatives **3**, **4**, **49**, and **51**. The obtained results concerning the monolaurin activity are particularly important because the compound is used as a key ingredient in various antimicrobial food additives. The results confirmed its high antibacterial potential. Two honeybee larval food fatty acid reference compounds, 10-HDA and sebacic acid, inhibited the *P. larvae* strains much more weakly (MIC 6400 μM) than other active compounds, and the same activity was observed for the monomethyl sebacate **23** (MIC 6400 μM). The MIC of 10-HDA correlated well with those that we had determined previously [16]. The reference antibiotics used for confirming the sensitivity of experimental *P. larvae* strains, ciprofloxacin and tylosin tartrate, were shown to be 50–100 times more antibacterially potent than the most efficient synthetic glycolipid derivative, dodecyl thiomannoside **4**.

In this study, a comparison of the antibacterial effect of glycolipid mimetics etherified either at the C-1 or C-6 position of the saccharide was performed for the first time. It showed that the antibacterial activity of some derivatives may be affected by the ether linkage position. In our case, dodecyl glucoside **6** (C-1 ether) exhibited lower efficacy than the glucoside **51** dodecyl etherified at C-6. The study further demonstrated that (thio)glycosides inhibited the Gram-positive strains of honeybee larval pathogen *P. larvae* with different efficacy. In our previous studies [52,53], other Gram-positive strains of *S. aureus* and *E. faecalis* were susceptible to some of these (thio)glycosides indicating that the nature of the saccharide units may affect their antibacterial efficacy. In line with the results of Smith et al. [49], methyl 6-*O*-dodecyl α-d-glucopyranoside **51** was a better inhibitor of the Gram-positive strain than its 6-*O*-lauroylated counterpart **52**. Here, the same effect was observed for the mannoside analogs **49** and **50**. Moreover, our observation that an optimal alkyl chain length for reaching a higher efficiency is C12 correlates with previous reports [49,53,54,66].

A very important result of this work was the identification of several compounds (**3**, **4**, **7**, **49**, **50**, **51**, and **52**) showing high efficacies against *P. larvae.* Their activities were either the same or 2–4 times higher (in dependence on tested *P. larvae* strain) than that of the reference compounds, lauric acid and monolaurin, and up to 128–356 times higher than the efficacies of 10-HDA and sebacic acid, the reference larval food compounds. Our previous findings suggested that the major RJ fatty acid 10-HDA together with abundant sebacic acid and other RJ fatty acids and proteins with anti-*P. larvae* properties could play a significant role in conferring antipathogenic activity to larval food and thus contribute to the resistance of individual larvae to *P. larvae* [16]. We assume that in honeybee colonies showing a higher resistance to AFB, the joint action of the antipathogenic compounds in the midguts of many infected young larvae may reach such high potency that this protects them from AFB. Such situations probably occur less frequently in the infected larvae in colonies showing a lower resistance to AFB. In this context, the fact that the most potent compounds identified were so many times more effective against *P. larvae* than the 10-HDA and sebacic acid was very important. Indeed, this suggests that the incorporation of only small amounts of any of these compounds into larval food could significantly contribute to increasing the constitutive anti-*P. larvae* activity of the food. Whether this increase could occur and whether it would suffice to provide larvae protection against AFB remains unknown and further research will be required to clarify this. We suppose that an efficient compound could mediate adequate antipathogenic action if: (1) it is not toxic or harmful to larvae or bees; (2) a suitable diet will be found in which it may be incorporated in adequate amounts; (3) it will be incorporated through nurse bees from the diet into larval food in such amounts that will contribute to the resistance of larvae to AFB.

## 3. Materials and Methods

### 3.1. General

Thin layer chromatography (TLC) was performed on aluminium sheets precoated with silica gel 60 F_254_ from Merck (Darmstadt, Germany). Flash column chromatography was carried out on silica gel 60 (0.040–0.060 mm) from Merck (Darmstadt, Germany) with distilled solvents (hexanes, ethyl acetate, chloroform, methanol). The anhydrous solvents (dichloromethane, methanol, DMF, and pyridine), monolaurin, lauric acid, sebacic acid, and monomethyl sebacate were purchased from Aldrich. 10-HDA with a 98% purity was purchased from AK Scientific, Inc. (Union City, CA, USA), and ciprofloxacin from Salutas Pharma GmbH (Barleben, Germany). Tylosin tartrate was obtained as a Tylancel veterinary preparation from Bares (Nitra, Slovakia). All reactions containing sensitive reagents were carried out under an argon atmosphere. ^1^H NMR and ^13^C NMR spectra were recorded at 25 °C with a Bruker AVANCE III HD 400 spectrometer. Chemical shifts were referenced to either TMS (δ 0.00, CDCl_3_ for ^1^H) or HOD (δ 4.87, CD_3_OD for ^1^H), and an internal CDCl_3_ (δ 77.00) or CD_3_OD (δ 49.00) for ^13^C. Optical rotations were measured on a Jasco P2000 polarimeter at 20 °C. High-resolution mass determination was performed by electrospray ionisation mass spectrometry (ESI-MS) on a Thermo Scientific Orbitrap Exactive instrument operating in positive mode. All the tested compounds were lyophilised before their use.

### 3.2. Synthesis

**Decyl 2,3,4,6-tetra-*O*-acetyl-1-thio-β-d-glucopyranoside (12).**

To a stirred solution containing 1,2,3,4,6-penta-*O*-acetyl-d-glucopyrannose (1.5 g, 3.84 mmol) in anhydrous CH_2_Cl_2_ (15 mL), 1-decanethiol (0.8 g, 0.97 mL, 4.6 mmol) was added. The reaction mixture was stirred for 20 min, cooled down on an ice bath, and BF_3_OEt_2_ (0.82 g, 0.73 mL, 5.76 mmol) was added dropwise. The resulting mixture was stirred for 15 min, brought to rt, and stirred for 2 h. The reaction mixture was diluted with CH_2_Cl_2_ (100 mL) and poured into ice-cold water (150 mL) under stirring. The organic phase was separated, washed with saturated aqueous NaHCO_3_ (3 × 75 mL), water (70 mL), dried (Na_2_SO_4_), filtered, and concentrated. Purification by column chromatography (hexane:EtOAc 7:1→3:1) gave **12**. Yield 0.66 g, 34%, yellowish oil, [α]_D_—31.2 (c 0.5, CHCl_3_). Analytical data are in agreement with Szabo et al. [67]. HRMS (ESI) *m/z*: calcd for C_24_H_41_O_9_S [M+H]^+^: 521.2376; found: 521.2415.

**General procedure for thioglycoside oxidation (Method A).**

To a stirred and 0 °C precooled solution containing corresponding thioglycoside (0.3 mmol) in CH_2_Cl_2_ (10 mL) *m*CPBA (0.9 mmol) was added. The reaction mixture was stirred at rt for 2 h, then diluted with CH_2_Cl_2_ (10 mL) and washed with saturated aqueous NaHCO_3_ (2 × 10 mL) and water (10 mL). The organic phase was dried (Na_2_SO_4_), filtered, and concentrated. The crude product was purified by column chromatography (hexane:EtOAc).

**Decyl 2,3,4,6-tetra-*O*-acetyl-α-d-mannopyranosyl sulfone (14).**

Treatment of **10** [53] (0.20 g, 0.38 mmol) as described in the general procedure (Method A) and purification by column chromatography (hexane:EtOAc 3:1→1:1) gave **14**. Yield 0.18 g, 85%, colourless oil. [α]_D_ + 33.8 (c 0.55, CHCl_3_). ^1^H NMR (400 MHz, CDCl_3_): δ 5.95 (dd, 1H, *J*_2,3_ 3.7 Hz, H-2), 5.59 (dd, 1H, *J* 9.1 Hz, H-3), 5.30 (t, 1H, *J*_4,5_ 9.4 Hz, H-4), 4.83 (d, 1H, *J* 2.3 Hz, H-1), 4.66 (ddd, 1H, H-5), 4.28 (dd, 1H, *J*_5,6b_ 5.6 Hz, *J*_6a,6b_ 12.5 Hz, H-6b), 4.15 (dd, 1H, *J*_5,6a_ 2.4 Hz, H-6a), 3.11 (ddd, 2H, *J* 2.6 Hz, *J* 6.5 Hz, *J* 9.5 Hz, SO_2_CH_2_), 2.16, 2.10, 2.07, 2.01 (each s, each 3H, 4 × OCOC*H*_3_), 1.92–1.25 (m, 16H, 8 × CH_2_), 0.88 (t, 3H, *J* 6.8 Hz, CH_3_). ^13^C NMR (100 MHz, CDCl_3_): δ 170.5, 169.8, 169.4, 169.4 (4 × O*C*OCH_3_), 87.8 (C-1), 73.6 (C-5), 69.0 (C-3), 65.5 (C-4), 65.0 (C-2), 62.6 (C-6), 51.1 (SO_2_CH_2_), 32.0, 29.6, 29.4, 29.3, 29.1, 28.7, 22.8, 21.7 (8 × CH_2_), 20.8(3×), 20.7 (4 × OCO*C*H_3_), 14.2 (CH_3_). HRMS (ESI) *m/z*: calcd for C_24_H_41_O_11_S [M+H]^+^: 553.2274; found: 553.2290.

**Dodecyl 2,3,4,6-tetra-*O*-acetyl-α-d-mannopyranosyl sulfone (15).**

The reaction was carried out according to the general procedure (Method A) with thiomannoside **11** [53] (0.20 g, 0.36 mmol). Column chromatography (hexane:EtOAc 3:1→1:1) gave **15**. Yield 0.17 g, 83%, colourless oil. [α]_D_ + 31.5 (c 0.82, CHCl_3_). ^1^H NMR (400 MHz, CDCl_3_): δ 5.95 (dd, 1H, *J* 3.7 Hz, H-2), 5.59 (dd, 1H, *J* 9.1Hz, H-3), 5.29 (t, 1H, *J*_4,5_ 9.4 Hz, H-4), 4.83 (d, 1H, *J* 2.2 Hz, H-1), 4.66 (ddd, 1H, H-5), 4.28 (dd, 1H, *J*_5,6b_ 5.6 Hz, *J*_6a,6b_ 12.5 Hz, H-6b), 4.15 (dd, 1H, *J*_5,6a_ 2.4 Hz, H-6a), 3.11 (ddd, 2H, *J* 2.5 Hz, *J* 6.8 Hz, *J* 9.6 Hz, SO_2_CH_2_), 2.16, 2.09, 2.06, 2.01 (each s, each 3H, 4 × OCOC*H*_3_), 1.91–1.24 (m, 20H, 10 × CH_2_), 0.88 (t, 3H, *J* 6.8 Hz, CH_3_). ^13^C NMR (100 MHz, CDCl_3_): δ 170.5, 169.8, 169.4, 169.3 (4 × O*C*OCH_3_), 87.8 (C-1), 73.6 (C-5), 69.0 (C-3), 65.5 (C-4), 65.0 (C-2), 62.6 (C-6), 51.1 (SO_2_CH_2_), 32.0, 29.7(2×), 29.6, 29.5, 29.4, 29.1, 28.7, 22.8, 21.7 (10 × CH_2_), 20.8(3×), 20.7 (4 × OCO*C*H_3_), 14.2 (CH_3_). HRMS (ESI) *m/z*: calcd for C_26_H_45_O_11_S [M+H]^+^: 565.2677; found: 565.2695.

**Decyl 2,3,4,6-tetra-*O*-acetyl-β-d-glucopyranosyl sulfone (16).**

The reaction was carried out according to the general procedure (Method A) with thiomannoside **12** [53] (0.30 g, 0.59 mmol). Column chromatography (hexane:EtOAc 3:1→1:1) gave **16**. Yield 0.24 g, 75%, yellowish oil. [α]_D_—18.8 (c 0.6, CHCl_3_). ^1^H NMR (400 MHz, CDCl_3_): δ 5.48 (t, 1H, *J*_2,3_ 9.6 Hz, H-2), 5.31 (t, 1H, *J*_3,4_ 9.3 Hz, H-3), 5.12 (t, 1H, H-4), 4.42 (d, 1H, *J*_1,2_ 9.9 Hz, H-1), 4.27 (dd, 1H, *J*_5,6b_ 4.9 Hz, *J*_6a,6b_ 12.6 Hz, H-6b), 4.20 (dd, 1H, *J*_5,6a_ 2.4 Hz, H-6a), 3.82 (ddd, 1H, *J*_4,5_ 10.1 Hz, H-5), 3.09 (dt, 2H, *J* 6.6 Hz, *J* 9.5 Hz, SO_2_CH_2_), 2.08, 2.06, 2.04, 2.02 (each s, each 3H, 4 × OCOC*H*_3_), 1.47–1.24 (m, 16H, 8 × CH_2_), 0.87 (t, 3H, *J* 6.8 Hz, CH_3_). ^13^C NMR (100 MHz, CDCl_3_): δ 170.5, 170.2, 169.5, 169.4 (4 × O*C*OCH_3_), 87.9 (C-1), 77.0 (C-5), 73.3 (C-3), 67.6 (C-4), 66.6 (C-2), 61.6 (C-6), 49.4 (SO_2_CH_2_), 32.0, 29.6, 29.4(2×), 29.3, 29.2, 28.7, 22.8 (8 × CH_2_), 20.9, 20.8(2×), 20.7 (4 × OCO*C*H_3_), 14.2 (CH_3_). HRMS (ESI) *m/z*: calcd for C_24_H_41_O_11_S [M+H]^+^: 537.2364; found: 537.2371.

**Dodecyl 2,3,4,6-tetra-*O*-acetyl-β-d-glucopyranosyl sulfone (17).**

The reaction was carried out according to the general procedure (Method A) with thiomannoside **13** [53] (0.35 g, 0.66 mmol). Column chromatography (hexane:EtOAc 4:1→2:1) gave **17**. Yield 0.30 g, 80%, yellowish oil. [α]_D_—20.1 (c 0.5, CHCl_3_). ^1^H NMR (400 MHz, CDCl_3_): δ 5.48 (t, 1H, dd, *J*_2,3_ 9.2 Hz, H-2), 5.31 (t, 1H, *J*_3,4_ 9.3 Hz, H-3), 5.12 (t, 1H, *J*_4,5_ 10.1 Hz, H-4), 4.42 (d, 1H, *J*_1,2_ 10.0 Hz, H-1), 4.27 (dd, 1H, *J*_5,6b_ 4.9 Hz, *J*_6a,6b_ 12.6 Hz, H-6b), 4.21 (dd, 1H, *J*_5,6a_ 2.5 Hz, H-6a), 3.82 (ddd, 1H, H-5), 3.09 (dt, 2H, *J* 6.6 Hz, *J* 9.5 Hz, SO_2_CH_2_), 2.09, 2.06, 2.04, 2.03 (each s, each 3H, 4 × OCOC*H*_3_), 1.92–1.78 (m, 2H, CH_2_), 1.46–1.24 (m, 18H, 9 × CH_2_), 0.87 (t, 3H, *J* 6.8 Hz, CH_3_). ^13^C NMR (100 MHz, CDCl_3_): δ 170.5, 170.2, 169.5, 169.4 (4 × O*C*OCH_3_), 87.9 (C-1), 77.0 (C-5), 73.3 (C-3), 67.6 (C-4), 66.6 (C-2), 61.6 (C-6), 49.4 (SO_2_CH_2_), 32.0, 29.7(2×), 29.6, 29.5, 29.4(2×), 29.2, 28.7, 22.8 (10 × CH_2_), 20.9, 20.8(2×), 20.7 (4 × OCO*C*H_3_), 14.3 (CH_3_). HRMS (ESI) *m/z*: calcd for C_26_H_45_O_11_S [M+H]^+^: 565.2677; found: 565.2689.

**General procedure for deprotection (Method B).**

The acetylated compound (0.25 mmol) was dissolved in anhydrous MeOH (9.5 mL), and MeONa (1M, 0.5 mL) was added. The reaction mixture was stirred for 16 h at rt, neutralized with Dowex 50 H^+^ form, filtered, and concentrated. The crude product was purified by column chromatography (EtOAc:MeOH).

**Decyl α-d-mannopyranosyl sulfone (18).**

The reaction was carried out according to the general procedure (Method B) with sulfone **14** (0.14 g, 0.25 mmol). Column chromatography (EtOAc:MeOH 0:1→7:1) gave **18**. Yield 76.5 mg, 82%, colourless oil. [α]_D_ + 47.5 (c 0.23, MeOH). ^1^H NMR (400 MHz, CD_3_OD): δ 4.95 (d, 1H, *J*_1,2_ 1.5 Hz, H-1), 4.54 (dd, 1H, *J*_2,3_ 3.8 Hz, H-2), 4.20–4.15 (m, 1H, H-5), 4.00 (dd, 1H, *J*_3,4_ 9.3 Hz, H-3), 3.92 (dd, 1H, *J*_5,6a_ 2.2 Hz, *J*_6a,6b_ 12.1 Hz, H-6a), 3.77–3.65 (m, 2H, H-4, H-6b), 3.27–3.23 (m, 2H, SO_2_CH_2_), 1.89–1.81 (m, 2H, CH_2_), 1.55–1.31 (m, 14H, 7 × CH_2_), 0.94 (t, 3H, *J* 6.8 Hz, CH_3_). ^13^C NMR (100 MHz, CD_3_OD): δ 92.8 (C-1), 79.8 (C-5), 72.8 (C-3), 67.6 (C-4), 66.8 (C-2), 63.1 (C-6), 51.1 (SO_2_CH_2_), 33.0, 30.6, 30.5, 30.4, 30.2, 29.6, 23.7, 22.5 (8 × CH_2_), 14.4 (CH_3_). HRMS (ESI) *m/z*: calcd for C_16_H_33_O_7_S [M+H]^+^: 369.1942; found: 369.1949.

**Dodecyl α-d-mannopyranosyl sulfone (19).**

The reaction was carried out according to the general procedure (Method B) with sulfone **15** (0.14 g, 0.25 mmol). Column chromatography (EtOAc:MeOH 0:1→7:1) gave **19**. Yield 79.3 mg, 80%, colourless oil. [α]_D_ + 52.3 (c 0.22, MeOH). ^1^H NMR (400 MHz, CD_3_OD): δ 4.95 (d, 1H, *J*_1,2_ 1.5 Hz, H-1), 4.53 (dd, 1H, *J*_2,3_ 3.7 Hz, H-2), 4.20–4.14 (m, 1H, H-5), 4.00 (dd, 1H, *J*_3,4_ 9.2 Hz, H-3), 3.92 (dd, 1H, *J*_5,6a_ 2.2 Hz, *J*_6a,6b_ 12.1 Hz, H-6a), 3.76–3.67 (m, 2H, H-4, H-6b), 3.27–3.22 (m, 2H, SO_2_CH_2_), 1.90–1.81 (m, 2H, CH_2_), 1.54–1.31 (m, 18H, 9 × CH_2_), 0.94 (t, 3H, *J* 6.8 Hz, CH_3_). ^13^C NMR (100 MHz, CD_3_OD): δ 92.8 (C-1), 79.8 (C-5), 72.8 (C-3), 67.7 (C-4), 66.8 (C-2), 63.2 (C-6), 51.1 (SO_2_CH_2_), 33.1, 30.6, 30.8, 30.7, 30.5, 30.5, 30.2, 29.6, 23.7, 22.5 (10 × CH_2_), 14.4 (CH_3_). HRMS (ESI) *m/z*: calcd for C_18_H_37_O_7_S [M+H]^+^: 397.2255; found: 397.2271.

**Decyl β-d-glucopyranosyl sulfone (20).**

The reaction was carried out according to the general procedure (Method B) with sulfone **16** (0.13 g, 0.25 mmol). Column chromatography (EtOAc:MeOH 0:1→ 6:1) gave **20**. Yield 74.6 mg, 81%, yellowish solid. [α]_D_—11.2 (c 0.51, MeOH). ^1^H NMR (400 MHz, CD_3_OD): δ 4.43 (d, 1H, *J*_1,2_ 9.5 Hz, H-1), 3.92 (dd, 1H, *J*_5,6a_ 2.1 Hz, H-6a), 3.80 (t, 1H, *J*_2,3_ 9.2 Hz, H-2), 3.71 (dd, 1H, *J*_5,6b_ 6.0 Hz, *J*_6a,6b_ 12.4 Hz, H-6b), 3.52–3.44 (m, 2H, H-3, H-5), 3.33–3.18 (m, 3H, H-4, SO_2_CH_2_), 1.90–1.82 (m, 2H, CH_2_), 1.54–1.32 (m, 14H, 7 × CH_2_), 0.93 (t, 3H, *J* 6.8 Hz, CH_3_). ^13^C NMR (100 MHz, CD_3_OD): δ 91.0 (C-1), 83.0 (C-5), 79.1 (C-3), 70.7, 70.6 (C-2, C-4), 62.6 (C-6), 52.0 (SO_2_CH_2_), 33.0, 30.6, 30.5, 30.4, 30.2, 29.6, 23.7, 22.2 (8 × CH_2_), 14.4 (CH_3_). HRMS (ESI) *m/z*: calcd for C_16_H_33_O_7_S [M+H]^+^: 369.1942; found: 369.1951.

**Dodecyl β-d-glucopyranosyl sulfone (21).**

The reaction was carried out according to the general procedure (Method B) with sulfone **17** (0.14 g, 0.25 mmol). Column chromatography (EtOAc:MeOH 0:1→ 6:1) gave **21**. Yield 81.7 mg, 83%, yellowish solid. [α]_D_—20.1 (c 0.23, MeOH). ^1^H NMR (400 MHz, CD_3_OD): δ 4.44 (d, 1H, *J*_1,2_ 9.5 Hz, H-1), 3.92 (dd, 1H, *J*_5,6a_ 2.1 Hz, H-6a), 3.80 (t, 1H, *J*_2,3_ 9.2 Hz, H-2), 3.71 (dd, 1H, *J*_5,6b_ 6.0 Hz, *J*_6a,6b_ 12.4 Hz, H-6b), 3.52–3.44 (m, 2H, H-3, H-5), 3.38–3.20 (m, 3H, H-4, SO_2_CH_2_), 1.90–1.83 (m, 2H, CH_2_), 1.54–1.31 (m, 18H, 9 × CH_2_), 0.94 (t, 3H, *J* 6.8 Hz, CH_3_). ^13^C NMR (100 MHz, CD_3_OD): δ 91.0 (C-1), 83.0 (C-5), 79.0 (C-3), 70.7, 70.6 (C-2, C-4), 62.6 (C-6), 52.0 (SO_2_CH_2_), 33.1, 30.7(2×), 30.6, 30.5(2×), 30.2, 29.6, 23.7, 22.2 (10 × CH_2_), 14.4 (CH_3_). HRMS (ESI) *m/z*: calcd for C_18_H_37_O_7_S [M+H]^+^: 397.2255; found: 397.2263.

**Decyl 1-thio-β-d-glucopyranoside (22).**

The reaction was carried out according to the general procedure (Method B) with thioglucoside **12** [67] (0.13 g, 0.25 mmol). Purification by column chromatography (EtOAc:MeOH 0:1→3:1) gave **22**. Yield 65 mg, 75%, yellowish oil, [α]_D—_36.8 (c 0.83, MeOH). Analytical data are in agreement with Szabo et al. [67] HRMS (ESI) *m/z*: calcd for C_16_H_33_O_5_S [M+H]^+^: 337.2043; found: 337.2048.

**Synthesis of acceptors 25, 26, and 28.**

The acceptors methyl 10-hydroxydecanoate (**25**) and methyl 12-hydroxydodecanoate (**26**) were prepared according to the reported procedure [57] and their analytical data are in agreement with Yamamoto et al. [68].

**(*E*)-methyl 10-hydroxydec-2-enoate (28).**

The solution of 10-HDA **27** (0.10 g, 0.539 mmol) in anhydrous methanol (8 mL) was stirred with ion-exchange resin (Amberlite IR 120, H^+^ from) for 4 days at rt. The resin was filtered off and the solvent was evaporated. Purification of the residue by column chromatography (hexane:EtOAc 4:1→2:1) gave **28**. Yield 78.5 mg, 73%, colorless oil. ^1^H NMR (400 MHz, CDCl_3_): δ 6.96 (dt, 1H, *J* 7.0 Hz, *J* 15.6 Hz, CH=), 5.81 (dt, 1H, *J* 1.6 Hz, *J* 15.6 Hz, CH=), 3.72 (s, 3H, OCH_3_), 3.63 (t, 2H, *J* 6.6 Hz, CH_2_OH), 2.21 (dd, 2H, *J* 1.6 Hz, *J* 7.1 Hz, CH_2_), 1.59–1.28 (m, 10H, 5 × CH_2_). ^13^C NMR (100 MHz, CDCl_3_): δ 167.3 (COOCH_3_), 149.8 (CH=), 121.0 (CH=), 51.5 (COO*C*H_3_), 63.1 (CH_2_OH), 32.8, 32.3, 29.3, 29.2, 28.1, 25.8 (7 × CH_2_). ^13^C NMR (400 MHz, CDCl_3_): δ 167.3 (*C*OOCH_3_), 149.8 (CH=), 121.0 (CH=), 63.1 (CH_2_OH), 51.5 (COO*C*H_3_), 32.8, 32.3, 29.3, 29.2, 28.1, 25.8 (6 × CH_2_). HRMS (ESI) *m/z*: calcd for C_11_H_20_O_3_Na [M+Na]^+^: 223.1305; found: 223.1310.

**General procedure for glycosylation (Method C).**

A mixture of trichloroacetimidate **29** [58] or **30** [59] (1.0 mmol), corresponding acceptor **25**, **26** or **28** (1.10 mmol), and 4 Å molecular sieves (100 mg/1 mmol of the donor) were stirred in anhydrous CH_2_Cl_2_ (10 mL) for 30 min. at rt. The reaction mixture was cooled to 0 °C and TMSOTf (0.10 mmol) was added. Then the reaction mixture was stirred for 20 min at rt. After neutralisation with solid NaHCO_3_, the solid was filtered off through Celite and rinsed with CH_2_Cl_2_ (15 mL). The solvent was evaporated and the residue was purified by column chromatography (hexane:EtOAc).

**Methyl 10-(2,3,4,6-tetra-*O*-benzoyl-α-d-mannopyranosyloxy)-decanoate (31).**

Treatment of glycosyl donor **29** with acceptor **25** as described in the general procedure (Method C) and purification by column chromatography (hexane:EtOAc 6:1→3:1) afforded **31**. Yield 0.56 g, 71%, colourless oil. [α]_D_—72.4 (c 0.96, CHCl_3_). ^1^H NMR (400 MHz, CDCl_3_): δ 8.10–8.05 (m, 4H, Harom), 7.97–7.95 (m, 2H, Harom), 7.85–7.82 (m, 2H, Harom), 7.61–7.25 (m, 12H, Harom), 6.09 (t, 1H, *J*_4,5_ 10.0 Hz, H-4), 5.92 (dd, 1H, *J*_2,3_ 3.3 Hz, *J*_3,4_ 10.1 Hz, H-3), 5.69 (dd, 1H, H-2), 5.08 (d, 1H, *J*_1,2_ 1.8 Hz, H-1), 4.69 (dd, 1H, *J*_5,6a_ 2.5 Hz, H-6a), 4.49 (dd, 1H, *J*_5,6b_ 4.6 Hz, *J*_6a,6b_ 12.1 Hz, H-6b), 4.42 (ddd, 1H, H-5), 3.82 (dt, 1H, *J* 6.8 Hz, *J* 9.6 Hz, OC*H*_2_(CH_2_)_8_), 3.66 (s, 3H, COOCH_3_), 3.57 (dt, 1H, *J* 6.5 Hz, *J* 9.5 Hz, OC*H*_2_(CH_2_)_8_), 2.31 (t, 2H, *J* 7.5 Hz, C*H*_2_COOCH_3_), 1.72–1.60 (m, 4H, 2 × CH_2_), 1.42–1.24 (m, 10H, 5 × CH_2_). ^13^C NMR (100 MHz, CDCl_3_): δ 174.5 (*C*OOCH_3_), 166.3, 165.7, 165.6, 165.5 (4 × C=O), 133.6, 133.5, 133.3, 133.2, 130.1, 130.0, 129.9, 129.8, 129.6, 129.3, 128.7, 128.6, 128.4 (Carom), 97.8 (C-1), 70.8 (C-2), 70.3 (C-3), 69.0, 68.9 (C-5, O*C*H_2_(CH_2_)_8_), 67.2 (C-4), 63.1 (C-6), 51.6 (COOCH_3_), 34.3, 29.6, 29.5(2×), 29.4, 29.3, 26.3, 25.1 (8 × CH_2_). HRMS (ESI) *m/z*: calcd for C_45_H_48_O_12_Na [M+Na]^+^: 803.3038; found: 803.3043.

**Methyl 12-(2,3,4,6-tetra-*O*-benzoyl-α-d-mannopyranosyloxy)-dodecanoate (32).**

Treatment of glycosyl donor **29** with acceptor **26** as described in the general procedure (Method C) and purification by column chromatography (hexane:EtOAc 6:1→3:1) afforded **32**. Yield 0.65 g, 79%, colourless oil. [α]_D_—47.7 (c 1.0, CHCl_3_). ^1^H NMR (400 MHz, CDCl_3_): δ 8.10–8.05 (m, 4H, Harom), 7.97–7.95 (m, 2H, Harom), 7.85–7.82 (m, 2H, Harom), 7.61–7.25 (m, 12H, Harom), 6.09 (t, 1H, *J*_4,5_ 10.0 Hz, H-4), 5.92 (dd, 1H, *J*_2,3_ 3.3 Hz, *J*_3,4_ 10.1 Hz, H-3), 5.69 (dd, 1H, H-2), 5.09 (d, 1H, *J*_1,2_ 1.7 Hz, H-1), 4.69 (dd, 1H, *J*_5,6a_ 2.5 Hz, H-6a), 4.49 (dd, 1H, *J*_5,6b_ 4.6 Hz, *J*_6a,6b_ 12.1 Hz, H-6b), 4.42 (ddd, 1H, H-5), 3.82 (dt, 1H, *J* 6.8 Hz, *J* 9.6 Hz, OC*H*_2_(CH_2_)_10_), 3.66 (s, 3H, COOCH_3_), 3.57 (dt, 1H, *J* 6.5 Hz, *J* 9.6 Hz, OC*H*_2_(CH_2_)_10_), 2.30 (t, 2H, *J* 7.6 Hz, C*H*_2_COOCH_3_), 1.74–1.60 (m, 4H, 2 × CH_2_), 1.44–1.25 (m, 14H, 7 × CH_2_). ^13^C NMR (100 MHz, CDCl_3_): δ 174.4 (*C*OOCH_3_), 166.3, 165.6(2×), 165.5 (4 × C=O), 133.6, 133.5, 133.3, 133.2, 130.0(2×), 129.9(2×), 129.8, 129.6, 129.3, 128.7, 128.6, 128.4 (Carom), 97.7 (C-1), 70.8 (C-2), 70.3 (C-3), 69.0(2×) (C-5, O*C*H_2_(CH_2_)_10_), 67.2 (C-4), 63.1 (C-6), 51.6 (COOCH_3_), 34.2, 29.7(2×), 29.6, 29.5(2×), 29.4, 29.3, 26.3, 25.1 (10 × CH_2_). HRMS (ESI) *m/z*: calcd for C_47_H_52_O_12_Na [M+Na]^+^: 831.3351; found: 831.3356.

**(*E*)-Methyl 10-(2,3,4,6-tetra-*O*-benzoyl-α-d-mannopyranosyloxy)dec-2-enoate (33).**

Reaction of glycosyl donor **29** (0.25 g, 0.34 mmol) with acceptor **28** as described in the general procedure (Method C) and purification by column chromatography (hexane:EtOAc 6:1→3:1) afforded **33**. Yield 0.22 g, 85%, yellowish oil. [α]_D—_58.3 (c 0.66, CHCl_3_). ^1^H NMR (400 MHz, CDCl_3_): δ 8.10–8.05 (m, 4H, Harom), 7.97–7.93 (m, 2H, Harom), 7.86–7.81 (m, 2H, Harom), 7.62–7.28 (m, 12H, Harom), 6.99 (dt, 1H, *J* 7.0 Hz, *J* 15.7 Hz, CH=), 6.09 (t, 1H, *J*_4,5_ 10.0 Hz, H-4), 5.92 (dd, 1H, *J*_2,3_ 3.4 Hz, *J*_3,4_ 10.1 Hz, H-3), 5.84 (dt, *J* 1.6 Hz, *J* 15.6 Hz, CH=), 5.69 (dd, 1H, H-2), 5.08 (d, 1H, *J*_1,2_ 1.8 Hz, H-1), 4.70 (dd, 1H, *J*_5,6a_ 2.6 Hz, H-6a), 4.45 (dd, 1H, *J*_5,6b_ 4.6 Hz, *J*_6a,6b_ 12.0 Hz, H-6b), 4.42 (ddd, 1H, H-5), 3.82 (dt, 1H, *J* 6.7 Hz, *J* 9.7 Hz, OC*H*_2_(CH_2_)_6_), 3.72 (s, 3H, COOCH_3_), 3.57 (dt, 1H, *J* 6.7 Hz, *J* 9.7 Hz, OC*H*_2_(CH_2_)_6_), 2.25–2.19 (m, 2H, CH_2_), 1.74–1.66 (m, 2H, CH_2_), 1.50–1.12 (m, 8H, 4 × CH_2_). ^13^C NMR (100 MHz, CDCl_3_): δ 167.3, 166.4, 166.0, 165.3, 165.2 (5 × C=O), 149.8 (CH=), 133.6, 133.3, 133.2, 130.0(2×), 129.9, 129.2, 128.7, 128.6, 128.4 (Carom), 121.0 (CH=), 97.8 (C-1), 70.8 (C-2), 70.3 (C-3), 69.0, 68.9 (C-5, O*C*H_2_(CH_2_)_6_), 67.2 (C-4), 63.1 (C-6), 51.5 (COO*C*H_3_), 32.4, 29.5, 29.3, 29.2, 28.1, 26.2 (6 × CH_2_). HRMS (ESI) *m/z*: calcd for C_47_H_50_O_12_Na [M+Na]^+^: 829.3194; found: 829.3202.

**Methyl 10-(2,3,4,6-tetra-*O*-benzoyl-β-d-glucopyranosyloxy)-decanoate (34).**

Reaction of glycosyl donor **30** with acceptor **25** as described in the general procedure (Method C) and purification by column chromatography (hexane:EtOAc 6:1→3:1) afforded **34**. Yield 0.56 g, 71%, yellowish oil. [α]_D_—6.7 (c 0.78, CHCl_3_). ^1^H NMR (400 MHz, CDCl_3_): δ 8.04–7.99 (m, 2H, Harom), 7.97–7.94 (m, 2H, Harom), 7.91–7.88 (m, 2H, Harom), 7.84–7.82 (m, 2H, Harom), 7.55–7.28 (m, 12H, Harom), 5.90 (t, 1H, *J*_3,4_ 9.7 Hz, H-3), 5.67 (t, 1H, *J*_4,5_ 9.7 Hz, H-4), 5.51 (dd, 1H, *J*_2,3_ 7.8 Hz, H-2), 4.83 (d, 1H, *J*_1,2_ 7.9 Hz, H-1), 4.63 (dd, 1H, *J*_5,6a_ 3.4 Hz, *J*_6a,6b_ 12.1 Hz, H-6a), 4.51 (dd, 1H, *J*_5,6b_ 5.2 Hz, H-6b), 4.16 (ddd, 1H, H-5), 3.91 (1H, *J* 6.2 Hz, *J* 9.7 Hz, OC*H*_2_(CH_2_)_8_), 3.66 (s, 3H, COOCH_3_), 3.53 (dt, 1H, *J* 6.5 Hz, *J* 9.5 Hz, OC*H*_2_(CH_2_)_8_), 2.31 (t, 2H, *J* 7.5 Hz, CH_2_), 1.56–1.49 (m, 4H, 2 × CH_2_), 1.21–1.06 (m, 10H, 5 × CH_2_). ^13^C NMR (100 MHz, CDCl_3_): δ 174.4 (*C*OOCH_3_), 166.3, 166.0, 165.4, 165.2 (4 × C=O), 133.5, 133.4, 133.30, 133.2, 130.0, 129.9, 129.8, 129.6, 129.0, 128.6, 128.5, 128.4 (Carom), 101.5 (C-1), 73.1 (C-3), 72.3, 72.1 (C-2, C-5), 70.5 (O*C*H_2_(CH_2_)_8_), 70.1 (C-4), 63.4 (C-6), 51.6 (COOCH_3_), 34.2, 29.5, 29.4, 29.3, 29.2(2×), 25.9, 25.1 (8 × CH_2_). HRMS (ESI) *m/z*: calcd for C_45_H_48_O_12_Na [M+Na]^+^: 803.3038; found: 803.3041.

**Methyl 12-(2,3,4,6-tetra-*O*-benzoyl-β-d-glucopyranosyloxy)-dodecanoate (35).**

Reaction of glycosyl donor **30** with acceptor **26** as described in the general procedure (Method C) and purification by column chromatography (hexane:EtOAc 6:1→3:1) afforded **35**. Yield 0.53 g, 65%, yellowish oil. [α]_D_—40.3 (c 0.78, CHCl_3_). ^1^H NMR (400 MHz, CDCl_3_): δ 8.03–8.00 (m, 2H, H arom), 7.97–7.94 (m, 2H, Harom), 7.91–7.88 (m, 2H, Harom), 7.84–7.81 (m, 2H, Harom), 7.54–7.28 (m, 12H, Harom), 5.90 (t, 1H, *J*_3,4_ 9.7 Hz, H-3), 5.67 (t, 1H, *J*_4,5_ 9.7 Hz, H-4), 5.51 (dd, 1H, *J*_2,3_ 7.8 Hz, H-2), 4.83 (d, 1H, *J*_1,2_ 7.9 Hz, H-1), 4.63 (dd, 1H, *J*_5,6a_ 3.4 Hz, *J*_6a,6b_ 12.1 Hz, H-6a), 4.51 (dd, 1H, *J*_5,6b_ 5.2 Hz, H-6b), 4.17 (ddd, 1H, H-5), 3.91 (1H, *J* 6.3 Hz, *J* 9.7 Hz, OC*H*_2_(CH_2_)_10_), 3.66 (s, 3H, COOCH_3_), 3.54 (dt, 1H, *J* 6.5 Hz, *J* 9.6 Hz, OC*H*_2_(CH_2_)_10_), 2.30 (t, 2H, *J* 7.5 Hz, CH_2_), 1.65–1.55 (m, 4H, 2 × CH_2_), 1.31–1.08 (m, 14H, 7 × CH_2_). ^13^C NMR (100 MHz, CDCl_3_): δ 174.5 (*C*OOCH_3_), 166.3, 166.0, 165.4, 165.2 (4 × C=O), 133.5, 133.4, 133.3, 133.2, 130.0, 129.9, 129.8, 129.6, 129.0, 128.6, 128.5, 128.4 (Carom), 101.5 (C-1), 73.1 (C-3), 72.3, 72.1 (C-2, C-5), 70.5 (O*C*H_2_(CH_2_)_10_), 70.1 (C-4), 63.4 (C-6), 51.6 (COO*C*H_3_), 34.3, 29.6(2×), 29.5, 29.4, 29.3, 29.2(2×), 25.9, 25.1 (10 × CH_2_). HRMS (ESI) *m/z*: calcd for C_47_H_52_O_12_Na [M+Na]^+^: 831.3351; found: 831.3359.

**(*E*)-Methyl 10-(2,3,4,6-tetra-*O*-benzoyl-β-d-glucopyranosyloxy)dec-2-enoate (36).**

Reaction of glycosyl donor **30** (0.25 g, 0.34 mmol) with acceptor **28** as described in the general procedure (Method C) and purification by column chromatography (hexane:EtOAc 6:1→3:1) afforded **36**. Yield 0.19 g, 71%, oil. [α]_D_—20.8 (c 0.65, CHCl_3_). ^1^H NMR (400 MHz, CDCl_3_): δ 8.03–7.99 (m, 2H, Harom), 7.97–7.95 (m, 2H, Harom), 7.91–7.89 (m, 2H, Harom), 7.84–7.81 (m, 2H, Harom), 7.56–7.27 (m, 12H, Harom), 6.91 (dt, 1H, *J* 7.0 Hz, *J* 15.6 Hz, CH=), 5.90 (t, 1H, *J*_3,4_ 9.7 Hz, H-3), 5.77 (dt, *J* 1.6 Hz, *J* 15.6 Hz, CH=), 5.67 (t, 1H, H-4), 5.52 (dd, 1H, *J*_2,3_ 9.8 Hz, H-2), 4.83 (d, 1H, *J*_1,2_ 7.8 Hz, H-1), 4.63 (dd, 1H, *J*_5,6a_ 3.3 Hz, H-6a), 4.51 (1H, *J*_5,6b_ 5.2 Hz, *J*_6a,6b_ 12.1 Hz, H-6b), 4.15 (ddd, 1H, *J*_4,5_ 9.5 Hz, H-5), 3.91 (dt, 1H, *J* 6.1 Hz, *J* 9.7 Hz, OC*H*_2_(CH_2_)_6_), 3.73 (s, 3H, COOCH_3_), 3.53 (dt, 1H, *J* 6.6 Hz, *J* 9.8 Hz, OC*H*_2_(CH_2_)_6_), 2.11–2.05 (m, 2H, CH_2_), 1.54–1.44 (m, 2H, CH_2_), 1.34–1.04 (m, 10H, 5 × CH_2_). ^13^C NMR (100 MHz, CDCl_3_): δ 167.3, 166.3, 166.0, 165.4, 165.2 (5 × C=O), 149.8 (CH=), 133.6, 133.4, 133.2, 130.0, 129.9(2×), 129.8, 129.6, 129.0, 129.0, 128.5, 128.5, 128.4 (Carom), 120.9 (CH=), 101.5 (C-1), 73.1 (C-3), 72.3 (C-5), 72.1 (C-2), 70.4 (O*C*H_2_(CH_2_)_6_), 70.0 (C-4), 63.4 (C-6), 51.5 (COO*C*H_3_), 32.3, 29.5, 29.1(2×), 28.0, 25.8 (6 × CH_2_). HRMS (ESI) *m/z*: calcd for C_47_H_50_O_12_Na [M+Na]^+^: 829.3194; found: 829.3200.

**General procedure for deprotection (Method D).**

To a protected glycoside (0.5 g) in methanol (17 mL) 1M MeONa (340 µL) was added and the reaction was stirred at rt for 1 h. The solvent was evaporated. Water (2 mL) was added and THF was dropped into the cloudy solution until it became clear. LiOH (170 mg) was added and the reaction mixture was stirred at rt for 1 h (TLC EtOAc:MeOH 4:1) and neutralized with Amberlite IR120 (H^+^ form) to pH 6. The resin was filtered off, rinsed with THF and the solvent was evaporated. The residue was diluted with water (20 mL), freeze-dried (2×) from water (2 × 20mL), and purified by column chromatography (CHCl_3_:MeOH).

**10-(α-d-Mannopyranosyloxy)-decanoic acid (37).**

Treatment of **31** as described in the general procedure (Method D) and purification by column chromatography (CHCl_3_:MeOH 9:1→3:1) afforded **37**. Yield 0.20 g, 92%, colourless oil. [α]_D_ + 25.1 (c 0.58, MeOH). ^1^H NMR (400 MHz, CD_3_OD): δ 4.73 (d, 1H, *J*_1,2_ 1.7 Hz, H-1), 3.82 (dd, 1H, *J*_5,6a_ 2.4 Hz, *J*_6a,6b_ 11.7 Hz, H-6°), 3.78 (dd, 1H, *J*_2,3_ 3.3 Hz, H-2), 3.75–3.68 (m, 3H, H-3, H-6b, OC*H*_2_(CH_2_)_8_), 3.61 (t, 1H, *J*_3,4_ 9.5 Hz, *J*_4,5_ 9.5 Hz, H-4), 3.55–3.50 (m, 1H, H-5), 3.41 (dt, 1H, *J* 6.3 Hz, *J* 9.6 Hz, OC*H*_2_(CH_2_)_8_), 2.24 (t, 2H, *J* 7.5 Hz, C*H*_2_COOH), 1.63–1.56 (m, 4H, 2 × CH_2_), 1.40–1.30 (m, 10H, 5 × CH_2_). ^13^C NMR (100 MHz, CD_3_OD): δ 170.0 (COOH), 101.6 (C-1), 74.6 (C-5), 72.7, 72.3 (C-2, C-3), 68.7(2×) (C-4, O*C*H_2_(CH_2_)_8_COOH), 62.9 (C-6), 35.0, 29.2(2×), 29.1, 29.0(2×), 25.9, 25.3 (8 × CH_2_). HRMS (ESI) *m/z*: calcd for C_16_H_30_O_8_Na [M+Na]^+^: 373.1833; found: 373.1843.

**12-(α-d-Mannopyranosyloxy)-dodecanoic acid (38).**

Treatment of **32** as described in the general procedure (Method D) and purification by column chromatography (CHCl_3_:MeOH 9:1→3:1) afforded **38**. Yield 0.21 g, 91%, colourless oil. [α]_D_ + 25.5 (c 0.6, MeOH). ^1^H NMR (400 MHz, CD_3_OD): δ 4.72 (d, 1H, *J*_1,2_ 1.7 Hz, H-1), 3.81 (dd, 1H, *J*_5,6a_ 2.4 Hz, *J*_6a,6b_ 11.7 Hz, H-6°), 3.77 (dd, 1H, *J*_2,3_ 3.3 Hz, H-2), 3.74–3.66 (m, 3H, H-3, H-6b, OC*H*_2_(CH_2_)_10_), 3.61 (t, 1H, *J*_3,4_ 9.5 Hz, *J*_4,5_ 9.5 Hz, H-4), 3.54–3.50 (m, 1H, H-5), 3.40 (dt, 1H, *J* 6.3 Hz, *J* 9.6 Hz, OC*H*_2_(CH_2_)_10_), 2.23 (t, 2H, *J* 7.5 Hz, C*H*_2_COOH), 1.61–1.54 (m, 4H, 2 × CH_2_), 1.37–1.28 (m, 14H, 7 × CH_2_). ^13^C NMR (100 MHz, CD_3_OD): δ 169.9 (COOH), 101.5 (C-1), 74.6 (C-5), 72.7, 72.3 (C-2, C-3), 68.6(2×) (C-4, OC*H*_2_(CH_2_)_10_), 62.9 (C-6), 36.2, 30.6(3×), 30.5(2×), 30.4, 29.4, 27.3, 26.6 (10 × CH_2_). HRMS (ESI) *m/z*: calcd for C_18_H_34_O_8_Na [M+Na]^+^: 401.2146; found: 401.2152.

**(*E*)-10-(α-d-Mannopyranosyloxy)-dec-2-enoic acid (39).**

Treatment of **33** (0.20 g, 0.26 mmol) as described in the general procedure (Method D) and purification by column chromatography (CHCl_3_:MeOH 9:1→3:1→1:2) afforded **39**. Yield 72 mg, 81%, oil. [α]_D_ + 15.5 (c 0.32, MeOH). ^1^H NMR (400 MHz, CD_3_OD): δ 6.74 (dt, 1H, *J* 7.0 Hz, *J* 15.6 Hz, CH=), 5.81 (dt, 1H, *J* = 1.5 Hz, *J* 15.5 Hz, CH=), 4.73 (d, 1H, *J*_1,2_ 1.7 Hz, H-1), 3.83 (dd, 1H, *J*_5,6a_ 2.4 Hz, *J*_6a,6b_ 11.7 Hz, H-6°), 3.78 (dd, 1H, *J*_2,3_ 3.4 Hz, H-2), 3.75–3.67 (m, 3H, H-3, H-6b, OC*H*_2_(CH_2_)_6_), 3.59 (t, 1H, *J*_3,4_ 9.5 Hz, *J*_4,5_ 9.5 Hz, H-4), 3.52 (ddd, *J*_5,6b_ 5.7 Hz, H-5), 3.42 (dt, 1H, *J* 6.3 Hz, *J* 9.7 Hz, OC*H*_2_(CH_2_)_6_), 2.20–2.14 (m, 2H, CH_2_), 1.61–1.28 (m, 10H, 5 × CH_2_). ^13^C NMR (100 MHz, CD_3_OD): δ 170.2 (COOH), 146.6 (CH=), 126.8 (CH=), 101.6 (C-1), 74.6 (C-5), 72.7, 72.3 (C-2, C-3), 68.7, 68.6 (C-4, O*C*H_2_(CH_2_)_6_), 62.9 (C-6), 33.0, 30.6, 30.3, 30.2, 29.8, 29.5 (6 × CH_2_). HRMS (ESI) *m/z*: calcd for C_18_H_32_O_8_Na [M+Na]^+^: 399.1989; found: 399.1995.

**10-(β-d-Glucopyranosyloxy)-decanoic acid (40).**

Treatment of **34** as described in the general procedure (Method D) and purification by column chromatography (CHCl_3_:MeOH 9:1→2.5:1) afforded **40**. Yield 0.18 g, 81%, colourless oil. [α]_D_—5.8 (c 0.53, MeOH). ^1^H NMR (400 MHz, CD_3_OD): δ 4.26 (d, 1H, *J*_1,2_ 7.8 Hz, H-1), 4.92–4.85 (m, 2H, H-6°, OC*H*_2_(CH_2_)_8_), 3.68 (dd, 1H, *J*_5,6b_ 5.3 Hz, *J*_6a,6b_ 11.9 Hz, H-6b), 3.54 (dt, 1H, *J* 6.3 Hz, *J* 9.6 Hz, OC*H*_2_(CH_2_)_8_), 3.36–3.26 (m, 3H, H-3, H-4, H-5), 3.18 (dd, 1H, *J*_2,3_ 9.0 Hz, H-2), 2.23 (t, 2H, *J* 7.5 Hz, C*H*_2_COOH), 1.66–1.58 (m, 4H, 2 × CH_2_), 1.41–1.31 (m, 10H, 5 × CH_2_). ^13^C NMR (100 MHz, CD_3_OD): δ 170.1 (COOH), 104.4 (C-1), 78.2 (C-3), 77.9 (C-5), 75.1 (C-2), 71.7 (C-4), 70.9 (O*C*H_2_(CH_2_)_8_), 62.8 (C-6), 36.9, 30.8, 30.6(2×), 30.5(2×), 27.1, 26.8 (8 × CH_2_). HRMS (ESI) *m/z*: calcd for C_16_H_30_O_8_Na [M+Na]^+^: 373.1833; found: 373.1839.

**12-(β-d-Glucopyranosyloxy)-dodecanoic acid (41).**

Treatment of **35** as described in the general procedure (Method D) and purification by column chromatography (CHCl_3_:MeOH 9:1→2.5:1) afforded **41**. Yield 0.17 g, 72%, yellowish oil. [α]_D_—5.1 (c 0.51, MeOH). ^1^H NMR (400 MHz, CD_3_OD): δ 4.26 (d, 1H, *J*_1,2_ 7.8 Hz, H-1), 4.93–4.85 (m, 2H, H-6°, OC*H*_2_(CH_2_)_10_), 3.67 (dd, 1H, *J*_5,6b_ 5.3 Hz, *J*_6a,6b_ 11.9 Hz, H-6b), 3.54 (dt, 1H, *J* 6.3 Hz, *J* 9.6 Hz, OC*H*_2_(CH_2_)_10_), 3.35–3.26 (m, 3H, H-3, H-4, H-5), 3.18 (dd, 1H, *J*_2,3_ 9.0 Hz, H-2), 2.27 (t, 2H, *J* 7.4 Hz, C*H*_2_COOH), 1.66–1.58 (m, 4H, 2 × CH_2_), 1.41–1.31 (m, 14H, 7 × CH_2_). ^13^C NMR (100 MHz, CD_3_OD): δ 170.2 (COOH), 104.4 (C-1), 78.1 (C-3), 77.9 (C-5), 75.1 (C-2), 71.7 (C-4), 70.9 (O*C*H_2_(CH_2_)_10_), 62.8 (C-6), 35.3, 30.8, 30.7(2×), 30.6(2×), 30.4, 30.3, 27.1, 26.2 (10 × CH_2_). HRMS (ESI) *m/z*: calcd for C_18_H_34_O_8_Na [M+Na]^+^: 401.2146; found: 401.2150.

**(*E*)-10-(β-d-Glucopyranosyloxy)-dec-2-enoic acid (42).**

Treatment of **36** (0.17 g, 0.22 mmol) as described in the general procedure (Method D) and purification by column chromatography (CHCl_3_:MeOH 10:1→5:1→1:2) afforded **42**. Yield 57 mg, 75 %, oil. [α]_D_—3.2 (c 0.41, MeOH). ^1^H NMR (400 MHz, CD_3_OD): δ 6.86 (dt, 1H, *J* 7.0 Hz, *J* 15.6 Hz, CH=), 5.82 (dt, 1H, *J* 1.5 Hz, *J* 15.5 Hz, CH=), 4.26 (d, 1H, *J*_1,2_ 7.8 Hz, H-1), 3.92 (dt, 1H, *J* 6.7 Hz, *J* 9.5 Hz, OC*H*_2_(CH_2_)_6_), 3.87 (dd, 1H, *J*_5,6a_ 1.8 Hz, *J*_6a,6b_ 11.8 Hz, H-6°), 3.68 (dd, 1H, *J*_5,6b_ 5.3 Hz, *J*_6a,6b_ 11.9 Hz, H-6b), 3.55 (dt, 1H, *J* 6.7 Hz, *J* 9.5 Hz, OC*H*_2_(CH_2_)_6_), 3.36–3.27 (m, H-3, H-4, H-5), 3.18 (dd, 1H, *J*_2,3_ 9.0 Hz, H-2), 2.21 (dq, 2H, *J* 1.5 Hz, *J* 7.2 Hz, CH_2_), 1.68–1.61 (m, 2H, CH_2_), 1.50–1.36 (m, 8H, 4 × CH_2_). ^13^C NMR (100 MHz, CD_3_OD): δ 172.8 (COOH), 148.8 (CH=), 124.9 (CH=), 104.4 (C-1), 78.1 (C-3), 77.9 (C-5), 75.1 (C-2), 71.7 (C-4), 70.8 (O*C*H_2_(CH_2_)_6_), 62.8 (C-6), 33.0, 30.7, 30.3, 30.2, 29.4, 27.0 (6 × CH_2_). HRMS (ESI) *m/z*: calcd for C_18_H_32_O_8_Na [M+Na]^+^: 399.1989; found: 399.1996.

**Methyl 2,3,4-tri-*O*-benzyl-6-*O*-dodecanyl-α-d-mannopyranoside (45).**

Compound **43** [60] (0.21 g, 0.45 mmol) was dissolved in anhydrous DMF (3 mL), the solution was cooled to 0 °C and sodium hydride (60% in mineral oil, 21.6 mg, 0.9 mmol) was added. After 15 min, dodecyl bromide (0.17 g, 0.16 mL, 0.67 mmol) was added and the resulting mixture was brought to rt. The stirring was continued for 16 h. The reaction was quenched with methanol (2 mL), diluted with EtOAc (20 mL), and washed with water (5 × 10 mL). The organic phase was dried (Na_2_SO_4_), filtered, and concentrated. Purification by column chromatography (hexane:EtOAc 30:1→10:1) gave **45**. Yield 0.21 g, 75%, oil. [α]_D_ + 30.4 (c 0.22, CHCl_3_). ^1^H NMR (400 MHz, CDCl_3_): δ 7.39–7.27 (m, 15H, Harom), 4.93 (d, 1H, *J* 10.9 Hz, OC*H*_2_Ph), 4.76 (d, 1H, *J*_1,2_ 1.8 Hz, H-1), 4.72 (br s, 2H, OC*H*_2_Ph), 4.63–4.60 (m, 3H, 2 × OC*H*_2_Ph), 3.95 (dd, 1H, H-3), 3.87 (dd, 1H, *J*_3,4_ 9.4 Hz, H-4), 3.78 (dd, 1H, *J*_2,3_ 2.5 Hz, H-2), 3.71–3.65 (m, 3H, H-5, H-6a, H-6b), 3.54 (dt, 1H, *J* 6.6 Hz, *J* 9.3 Hz, OC*H*_2_(CH_2_)_10_CH_3_), 3.42 (dt, 1H, *J* 6.9 Hz, *J* 9.3 Hz, OC*H*_2_(CH_2_)_10_CH_3_), 3.31 (s, 3H, OCH_3_), 1.62–1.25 (m, 20H, 10 × CH_2_), 0.88 (t, 3H, *J* 6.8 Hz, CH_3_). ^13^C NMR (100 MHz, CDCl_3_): δ 138.9, 138.7, 138.5, 128.5, 128.4, 128.0, 128.0, 127.7(2×), 127.6 (Carom), 99.1 (C-1), 80.4 (C-4), 75.2(2×) (O*C*H_2_Ph, C-3), 74.7 (C-2), 72.6(2×) (2 × O*C*H_2_Ph), 71.9, 71.7 (O*C*H_2_(CH_2_)_10_CH_3_, C-5), 70.2 (C-6), 54.8 (OCH_3_), 32.1, 29.9, 29.8(3×), 29.7(2×), 29.5, 26.4, 22.8 (10 × CH_2_), 14.3 (CH_3_). HRMS (ESI) *m/z*: calcd for C_40_H_57_O_6_ [M+H]^+^: 633.4150; found: 633.4158.

**Methyl 2,3,4-tri-*O*-benzyl-6-*O*-lauroyl-α-d-mannopyranoside (46).**

To a solution of **43** (0.21 g, 0.45 mmol) in anhydrous pyridine (3 mL), DMAP (10 mg) and lauroyl chloride (0.20 g, 0.21 mL, 0.90 mmol) were added. The solution was stirred at rt for 24 h and then concentrated. Purification of the residue by column chromatography (hexane:EtOAc 30:1→8:1) gave **46**. Yield 0.23 g, 79%, oil. Analytical data are in agreement with Smith et al. [49].

**Methyl 2,3,4-tri-*O*-benzyl-6-*O*-dodecanyl-α-d-glucopyranoside (47).**

Treatment of **44** [61] (0.42 g, 0.9 mmol) by the same procedure as described for **45** gave **47** (0.54 g, 94 %), oil. ^1^H NMR (400 MHz, CDCl_3_): δ 7.37–7.27 (m, 15H, Harom), 4.97 (d, 1H, *J*_1,2_ 10.8 Hz, H-1), 4.88 (d, 1H, *J* 10.9 Hz, OC*H*_2_Ph), 4.82 (d, 1H, *J* 11.2 Hz, OC*H*_2_Ph), 4.79 (d, 1H, *J* 12.8 Hz, OC*H*_2_Ph), 4.66 (d, 1H, *J* 12.1 Hz, OC*H*_2_Ph), 4.62–4.58 (m, 2H, OC*H*_2_Ph), 3.98 (t, 1H, *J*_3,4_ 9.2 Hz, H-3), 3.73–3.45 (m, 6H, H-2, H-4, H-5, H-6a, H-6b, OC*H*_2_(CH_2_)_10_CH_3_), 3.36 (s, 3H, OCH_3_), 3.36–3.31 (m, 1H, OC*H*_2_(CH_2_)_10_CH_3_), 1.59–1.55 (m, 2H, CH_2_), 1.32–1.22 (m, 18H, 9 × CH_2_), 0.87 (t, 3H, *J* 6.8 Hz, CH_3_). HRMS (ESI) *m/z*: calcd for C_40_H_57_O_6_ [M+H]^+^: 633.4150; found: 633.4154.

**Methyl 2,3,4-tri-*O*-benzyl-6-lauroyl-α-d-glucopyranoside (48).**

Treatment of **44** (0.47 g, 1.02 mmol) by the same procedure as described for **46** gave **48** (0.61 g, 94 %), oil. Analytical data are in agreement with Smith et al. [49].

**Methyl 6-*O*-dodecanyl-α-d-mannopyranoside (49).**

To a solution of **45** (0.17 g, 0.27 mmol) in MeOH (20 mL) 10% Pd/C (0.1 g) was added. The reaction mixture was stirred under an H_2_ atmosphere for 4 h. The catalyst was filtered off and the filtrate was evaporated. The residue was purified by column chromatography (hexane:EtOAc 9:1→0:1) to afford **49**. Yield 77.2 mg, 79%, oil. [α]_D_ + 36.2 (c 0.46, CHCl_3_). ^1^H NMR (400 MHz, CDCl_3_): δ 4.73 (d, 1H, *J*_1,2_ 1.6 Hz, H-1), 3.92 (dd, 1H, *J*_2,3_ 3.0 Hz, H-2), 3.80–3.65 (m, 5H, H-3, H-4, H-5, H-6a, H-6b), 3.53 (dt, 1H, *J* 6.7 Hz, *J* 9.4 Hz, OC*H*_2_(CH_2_)_10_CH_3_), 3.49 (dt, 1H, *J* 6.9 Hz, *J* 9.4 Hz, OC*H*_2_(CH_2_)_10_CH_3_), 3.38 (s, 3H, OCH_3_), 3.20 (br s, 1H, OH), 2.86 (br s, 1H, OH), 2.63 (br s, 1H, OH), 1.62–1.55 (m, 2H, CH_2_), 0.87 (t, 3H, *J* 6.7 Hz, CH_3_). ^13^C NMR (100 MHz, CDCl_3_): δ 100.9 (C-1), 72.4 (O*C*H_2_(CH_2_)_10_CH_3_), 71.8(2×) (C-3, C-6), 70.6 (C-2), 70.5 (C-4), 69.3 (C-5), 55.2 (OCH_3_), 32.1, 29.8(3×), 29.7(2×), 29.6, 29.5, 26.2, 22.8 (10 × CH_2_), 14.3 (CH_3_). HRMS (ESI) *m/z*: calcd for C_19_H_39_O_6_ [M+H]^+^: 363.2741; found: 363.2745.

**Methyl 6-*O*-lauroyl-α-d-mannopyranoside (50).**

Treatment of **46** (0.27 g, 0.42 mmol) by the same procedure as described for **49** gave **50**. Yield 83.8 mg, 72%, oil. Analytical data are in agreement with Smith et al. [49].

**Methyl 6-*O*-dodecanyl-α-d-glucopyranoside (51).**

Treatment of **47** (0.54 g, 0.85 mmol) by the same procedure as described for **49** gave **51** (0.26 g, 82 %) white solid. Analytical data are in agreement with Smith et al. [49].

**Methyl 6-*O*-lauroyl-α-d-glucopyranoside (52).**

Treatment of **48** (0.55 g, 0.85 mmol) by the same procedure as described for **49** gave **52** (0.31 g, 98 %) white solid. Analytical data are in agreement with Smith et al. [49].

### 3.3. Biology

***Paenibacillus larvae* strains**.

The CCM 4483 strain with an ERIC I genotype and CCM 4486 with an ERIC II genotype were obtained from the Czech collection of microorganisms (CCM).

**Bacterial cultivation.**

The bacterial cultivations were performed in an MYPGP *P. larvae* cultivation medium [69]. The pH values of the media were adjusted to values of 6.6, i.e., lower than that of the original medium (7.2–7.4). The lower pH corresponded better with the pH conditions in the midguts of young larvae where *P. larvae* reproduce naturally [16]. The cultivations were performed at 35 °C, a temperature typical for honeybee hives.

**Determination of minimal inhibitory concentrations.**

A broth microdilution method in 96-well microplates was used to determine the MICs of the tested compounds. First, stock solutions with 20 mM concentrations of individual compounds were prepared in methanol and stored at -25 °C. Before performing the tests, working solutions with suitable concentrations of compounds were prepared from those using two-fold serial dilutions. Stock and working solutions of antibiotics were prepared in dimethyl sulfoxide (DMSO). Overnight bacterial cultures of the examined strains were prepared on an orbital shaker and diluted in a cultivation medium to a final concentration of 1 × 10^5^ CFU/mL. Aliquots of the diluted cultures (147 µL) were pipetted into the wells of sterile polystyrene microplates. Then, 3 µL of the working solutions of the tested compounds were added to the wells to reach final concentrations of the compounds and the antibiotics ranging from 12.5 µM to 6400 µM and 0.125–6.25 µM, respectively. Each compound was pipetted into wells in triplicate. The microplates were shaken on a microplate shaker Biofil (Merci, Paris, France) at 1200 rpm for 5 min and then left to incubate under stationary conditions for 43 h. The shaking of the microplates was repeated after 18 h and at the end of the cultivation. Bacterial growth was determined spectrophotometrically by measuring the absorbance at 630 nm using a Mithras^2^LB 943 microplate reader (Berthold Technologies). The positive and negative controls of bacterial growth contained 3 µL of methanol or DMSO. The antibacterial sensitivity of the used *P. larvae* strains was evaluated with ciprofloxacin and tylosin tartrate antibiotics. The MIC of each compound was determined by three independent tests.

## 4. Conclusions

This work is the first study evaluating the susceptibility of *P. larvae* strains of distinct ERIC genotypes to synthetic carbohydrate lipid-like compounds. These compounds consisted of non-toxic, biodegradable, and eco-friendly alkyl, fatty acid, and carbohydrate units. The study confirmed that the structure of the sugar units and the length of the alkyl chains had an impact on the antibacterial efficacy of the derivatives. The incorporation of an alkyl unit to the saccharide at the C-6 position by ether linkage was shown to be more beneficial than the ester function between these units. The thioglycosides were generally more active than their *O*-counterparts and sulfones. The C-1 saccharides conjugated with fatty acids, including 10-HDA, were shown to be inactive in the performed tests. This demonstrated that a polarity of the functional group terminating the alkyl chain was another important factor modulating the antibacterial effects of the sugar-based amphiphiles. It can be concluded that some carbohydrate-based amphiphiles with appropriate sugar cores and dodecyl alkyl chains may act as efficient inhibitors of the honeybee pathogen *P. larvae*. The most potent anti-*P. larvae* derivatives identified in this work represent potential candidates that could be examined for their ability to improve larval protection against AFB.

## Figures and Tables

**Table 1:** Antibacterial efficacy of carbohydrate lipid-like derivatives and the reference compounds against two *P. larvae* strains.

Compound		*P. larvae* CCM 4483 ^(a)^		*P. larvae* CCM 4486 ^(b)^	
		MIC (µM)	MIC (µg/mL)	MIC (µM)	MIC (µg/mL)
**1**	C10Man	400	128.2	200	64.1
**2**	SC10Man	100	33.6	50	16.8
**3**	C12Man	25	8.7	25	8.7
**4**	SC12Man	25	9.1	12.5	4.6
**5**	C10Glc	800	256.3	800	256.3
**22**	SC10Glc	400	134.6	400	134.6
**6**	C12Glc	100	34.8	50	17.4
**7**	SC12Glc	50	18.2	25	9.1
**8**	C10Gal	800	256.4	400	128.2
**9**	C12Gal	800	278.8	800	278.8
**18**	SO_2_C10Man	400	147.4	400	147.4
**19**	SO_2_C12Man	100	39.7	50	19.8
**20**	SO_2_C10Glc	800	294.8	400	147.4
**21**	SO_2_C12Glc	100	39.7	50	19.8
**37**	ManC10acid	>6400	>2242.6	>6400	>2242.6
**38**	ManC12acid	>6400	>2242.1	>6400	>2242.1
**39**	Man10-HDA	>6400	>2229.7	>6400	>2229.7
**40**	GlcC10acid	>6400	>2242.6	>6400	>2242.6
**41**	GlcC12acid	>6400	>2422.1	>6400	>2422.1
**42**	Glc10-HDA	>6400	>2229.7	>6400	>2229.7
**49**	MeMan6-Dod	25	9.1	25	9.1
**50**	MeMan6-Lau	50	18.8	50	18.8
**51**	MeGlc6-Dod	25	9.1	25	9.1
**52**	MeGlc6-Lau	50	18.8	50	18.8
**23**	**Monomethyl sebacate**	6400	1384.1	6400	1384.1
**27**	**10-HDA**	6400	1192	6400	1192
**Sebacic acid**		6400	1294.4	6400	1294.4
**Lauric acid**		50	10	50	10
**Monolaurin**		50	13.7	25	6.9
**Ciprofloxacin**		<0.25	<0.1	<0.25	<0.1
**Tylosin tartrate**		<0.25	<0.3	<0.25	<0.3

^(a)^ ERIC I strain, ^(b)^ ERIC II strain, reference compounds are highlighted by bold letters. MIC (minimal inhibitory concentration) was determined by three independent tests which provided the same result for each compound

## Data Availability

Not applicable.
